# The influence of sacrocolporectopexy on pelvic anatomy assessed in an upright position using MRI

**DOI:** 10.1111/codi.70114

**Published:** 2025-05-07

**Authors:** Mart C. P. Kortman, Jan W. P. Vanstiphout, Akeel Alhafidh, Frank F. J. Simonis, Anique T. M. Grob

**Affiliations:** ^1^ Multimodality Medical Imaging (M3i) Group, Technical Medical Centre University of Twente Enschede The Netherlands; ^2^ Department of Gynecology Ziekenhuisgroep Twente Hengelo The Netherlands; ^3^ Department of Surgery Ziekenhuisgroep Twente Hengelo The Netherlands; ^4^ Magnetic Detection and Imaging (MD&I) Group, Technical Medical Centre University of Twente Enschede The Netherlands

**Keywords:** Internal rectal prolapse, pelvic anatomy, pelvic organ prolapse, sacrocolporectopexy, upright magnetic resonance defaecography

## Abstract

**Aim:**

Rectopexy with concomitant sacrocolpopexy (sacrocolporectopexy) is the favoured technique for treating combined pelvic organ prolapse and internal or external rectal prolapse, despite limited functional improvement. Previous studies have assessed anatomical change after standalone rectopexy or sacrocolpopexy, based on supine MRI defaecography. Since a supine position can underestimate the extent of pelvic organ prolapse, it might also incorrectly assess the anatomical effect of sacrocolporectopexy. The aim of this study was to assess the effect of sacrocolporectopexy on the pelvic anatomy in an upright position.

**Method:**

Twenty one female patients undergoing sacrocolporectopexy from December 2022 to June 2024 were included. All patients underwent physical examination and MRI defaecography preoperatively and postoperatively. The descent of the bladder, vaginal vault and anorectal junction and the size of the rectocele and enterocele were assessed on the MRI defaecography images during maximum straining. Significance was tested using a paired *t*‐test and an improvement of ≥10 mm was considered clinically relevant. The results were compared with previous studies, which used supine assessment.

**Results:**

Postoperative improvement was found for the bladder, vaginal vault, anorectal junction, rectocele and enterocele with 14, 44, 5, 16 and 54 mm respectively. The bladder, vaginal vault, rectocele and enterocele showed clinically relevant improvement. Compared with supine results, upright assessments revealed a larger organ lift for the vaginal vault as well as a higher, overall, position of the anorectal junction.

**Conclusion:**

Upright assessment of sacrocolporectopexy differs from supine assessment, with statistical and clinically relevant lift for the pelvic organs.

**What does this paper add to the literature?**

There is increasing interest in the study of pelvic organ prolapse and its treatment in an upright position. This study concludes that there is a significant improvement of bladder prolapse, vaginal vault prolapse, rectocele and enterocele, which is the first step towards optimizing surgery with improved functional outcomes.

## INTRODUCTION

Faecal incontinence (FI) and obstructive defaecation syndrome (ODS) are known for their large negative impact on quality of life and the stigma surrounding these symptoms [1]. Symptoms of FI and ODS are often a result of pelvic organ prolapse (POP), more specifically external rectal prolapse (ERP), internal rectal prolapse (IRP), complex rectocele or enterocele [2, 3]. When conservative treatment fails, surgery by means of robot‐assisted or laparoscopic ventral mesh rectopexy (VMR) is indicated [4]. During the VMR procedure a polypropylene mesh implant is attached inferiorly to the anterior rectal wall and posterior fornix of the vagina and superiorly to the sacral promontory [5]. VMR is proven to be effective, with the (internal) rectal prolapse being resolved in more than 90% of the patients. However, a large retrospective cohort study showed that complaints of FI and ODS persist in 30%–40% of patients after VMR [6]. In recent years a shift in VMR procedures has been seen, with an increasing focus on combined surgery with the gynaecological sacrocolpopexy (SCP) procedure [7]. During this combined sacrocolporectopexy (SCRP) procedure an additional mesh is attached to the anterior vaginal wall and posterior bladder wall and fixed to the posterior mesh attached to the rectum.

Assessment of the pelvic anatomy and functionality is routinely done with conventional X‐ray, defaecography (CD) or dynamic magnetic resonance defaecography (MRD) [8]. To allow for clinical decision‐making, the limitations of all techniques should be considered. CD has a low soft‐tissue contrast as it only visualizes radio‐opaque structures, while MRD allows for visualization of soft tissue but is performed in a nonphysiological supine position [9]. To fully understand the anatomical effect of VMR, an imaging modality combining soft‐tissue assessment and a nonsupine position is required. Upright MRI offers both requirements and has been used in several studies within the field of POP, showing promising results and a striking difference between patients in supine and upright positions [10, 11, 12].

Available studies have only reported on the anatomical effect of standalone SCP or standalone VMR, not on the effect of SCRP. Thereby, they used supine MRD to assess the effect. Their results suggest that most of the anatomical change is visible in structures that are directly attached to the mesh implants during surgery which, for SCRP, are the bladder, the vaginal vault (VV), the anterior rectal wall (rectocele) and the rectovaginal septum (enterocele) [13, 14, 15]. However, upright assessment might show a different extent of the surgical effect. To test this hypothesis the aim of this study is to assess the anatomical effect of SCRP on the pelvic anatomy in the upright position.

## METHOD

In this prospective longitudinal study patients were selected from the specialized outpatient clinic for complex POP in the Ziekenhuisgroep Twente (ZGT) hospital in Hengelo, the Netherlands, between December 2022 and June 2024. Female patients were included in this study when an IRP or ERP was proven through CD, a POP was found through pelvic organ prolapse quantification (POP‐Q) staging and the patient opted for robotic SCRP. The exclusion criteria were previous pelvic reconstructive surgery using mesh implants, inability to stand for 20 min without assistance, patient not eligible to undergo an MRI scan, jeans size greater than 52 (EU) or 22 (US) due to limited MRI coil circumference, or insufficient knowledge of the Dutch language. All patients were aged ≥18 years and provided written informed consent. The study was approved by a medical ethics committee and registered as NL79717.091.21. Study size was not formally calculated due to its exploratory setting.

Pre‐ and postoperative measurements by means of POP‐Q, patient‐reported outcome measures (PROMs) and MR images were acquired in all patients. There was no set time limit between the preoperative measurements and the surgery. The postoperative measurements were performed after the first postoperative visit to the outpatient clinic, typically around 10 weeks after surgery. POP‐Q point Aa or Ba (whichever showed the most prolapse) was used to quantify the anterior vaginal wall prolapse, point C was used to quantify the apical prolapse and point Ap or Bp was used to quantify the posterior prolapse [16]. PROMs consisted of the Pelvic Floor Distress Inventory (PFDI)‐20, the Pelvic Floor Impact Questionnaire (PFIQ)‐7 and the Prolapse Incontinence Sexual Questionnaire (PISQ)‐12, which were acquired pre‐ and postoperatively, and the Patient Global Impression of Improvement (PGI‐I) which was only acquired postoperatively. Scoring 1 or 2 on the PGI‐I was considered an improvement over the preoperative situation.

Sagittal MR images were acquired with a 0.25 T tiltable, open‐configuration MR system (G‐Scan Brio, Esaote, Genoa, Italy). A dedicated multichannel spine coil was used for acquisition. Patients were prepared by filling the vagina with 30–50 mL of ultrasound gel and the rectum with at least 180 mL, dependent on the patient's sensation to defaecate, of stool‐mimicking instant mashed potatoes. Three‐dimensional (3D) hybrid contrast enhancement (HYCE) data were acquired in the sagittal direction for an overview of the anatomy, whereas single slice 2D HYCE data were acquired for dynamic imaging during defaecography. During the study, MRI sequence parameters of the 3D HYCE sequence were changed to improve the image quality. This did not affect the measurements that were included in the analysis. The sequence parameter settings and their values are reported in Table 1.

**TABLE 1:** Technical specification of magnetic resonance sequences. During the study, the three‐dimensional hybrid contrast enhancement (3D HYCE) sequence image quality was improved.

Parameter	3D HYCE sequence	2D HYCE sequence
Echo time	4 ms	3.5 ms
Repetition time	8 ms	7 ms
Flip angle^a^	60°/35°	70°
Field of view	250 mm × 250 × 164 mm	400 mm × 400 mm × 15 mm
Acquisition matrix^a^	224 × 124 × 64/224 × 170 × 50	208 × 208
Reconstructed resolution	0.5 mm × 0.5 mm × 0.5 mm	0.8 mm × 0.8 mm
Number of averages	3	2
Acquisition time^a^	3:32 min/3:41 min	1 s (per frame)

^a^ Parameter value changed after improvement. The second mentioned value was used to improve the image quality or changed due to other improved parameters.

Surgery was performed by a team of two experienced and specialized surgeons (one colorectal surgeon and one urogynaecologist). VMR was performed as described by D'Hoore and Penninckx and robotic assistance was by means of the Da Vinci Xi system (Intuitive Surgical, Sunnyvale, CA, USA) [5]. The patients were positioned in the steep Trendelenburg position and the POP, IRP and/or ERP was redressed. A heavyweight mesh (Bard® Mesh, BD, Franklin Lakes, NJ, USA) was attached to the ventral aspect of the rectum and posterior fornix of the vagina with nonabsorbable Ethibond (Johnson & Johnson, New Brunswick, NJ, USA) sutures. Thereafter SCP was started by dissecting the vesicovaginal septum to the level of the urethra, whereafter a lightweight mesh (Restorelle®, Coloplast, Humlebæk, Denmark) was attached to the anterior vaginal wall and posterior vesical wall with nonabsorbable Ethibond (Johnson & Johnson, New Brunswick, NJ, USA) sutures. The two meshes were attached to each other above the VV with nonabsorbable sutures. The cranial aspect of the mesh was attached to the sacral promontory with nonabsorbable Ethibond (Johnson & Johnson, New Brunswick, NJ, USA) sutures. The peritoneum was then closed using a V‐Loc™ self‐locking suture (Medtronic, Minneapolis, MN, USA).

**FIGURE 1:**
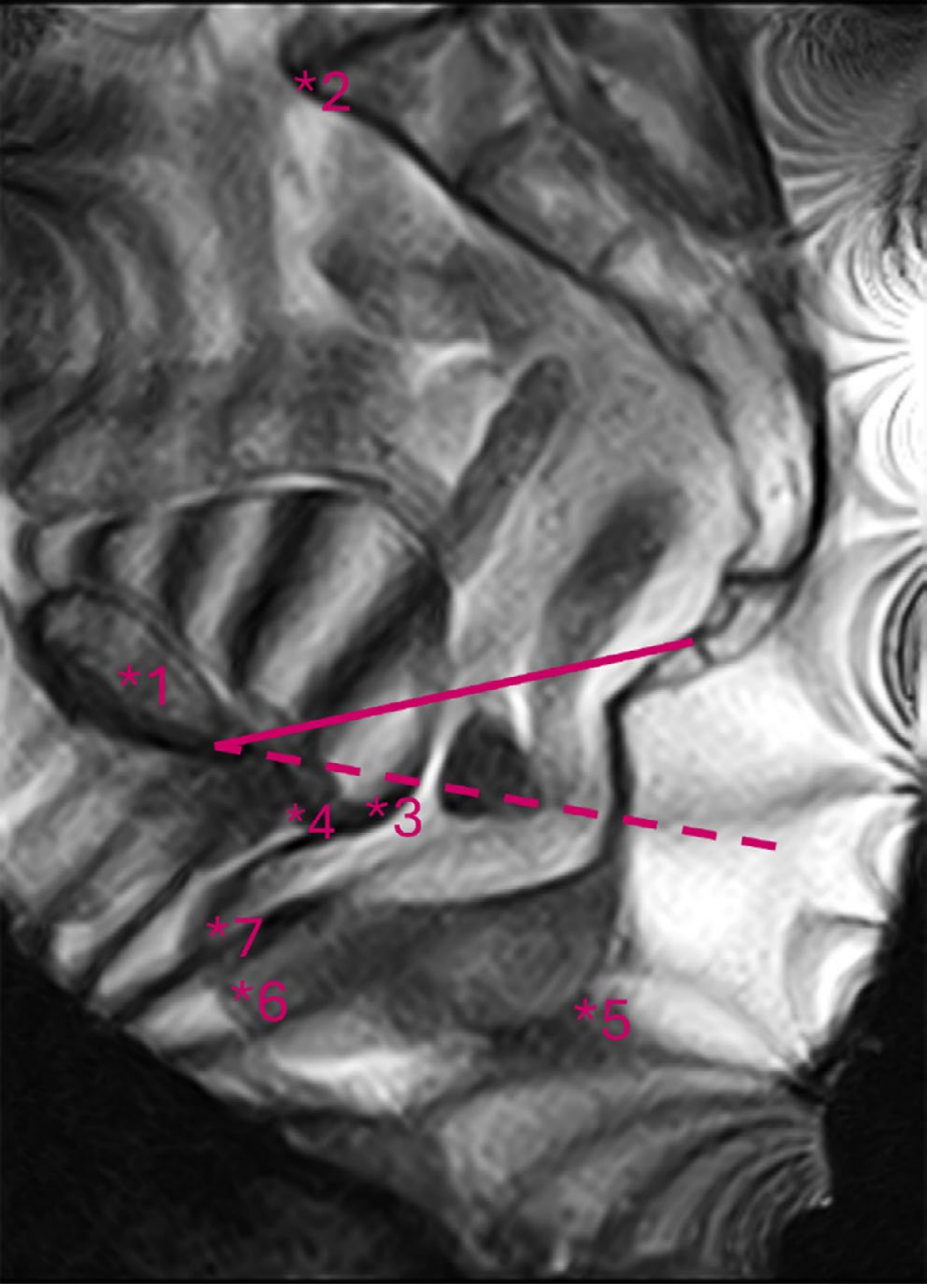
Anatomical landmarks and reference line used for analysis. The full line is the pubococcygeal line and the dashed line is the pelvic inclination correction system line. The asterisk in front of the number represents the position used for distance calculation. 1, pubic symphysis; 2, sacral promontory; 3, lowest point of bladder; 4, vaginal vault; 5, anorectal junction; 6, most anterior point of the rectum (rectocele); 7, lowest point of small intestines (enterocele).

MATLAB 2022b (MathWorks, Natick, MA, USA) was used to manually annotate anatomical landmarks in the rest and maximum strain frames acquired with the 2D HYCE sequence. The rest frame and the maximum strain frame were defined as the first frame of the sequence and the frame directly before defaecation. The upright pelvic inclination correction system (PICS) line, which is based on the inferior pubic point (IPP), sacrococcygeal joint and lowest coccygeal joint was used as a reference. A second reference line, the pubococcygeal line (PCL) was determined to allow for a direct comparison with previous literature [13, 14]. The shortest distance to these reference lines of the lowest point of the bladder, the highest point of the VV, the lowest point of the small intestines and the anorectal junction (ARJ) to the PICS were calculated. The M‐line, a measure for perineal descent, was defined as the distance from the ARJ to the PCL and the H‐line, a measure for hiatal widening, as the distance from the IPP to the ARJ. The distance between the line of the anterior anal canal and the most anterior point of the rectum was used as a measure for rectocele size. In Figure 1, the PICS (dashed line) and PCL (line) reference lines are depicted alongside the anatomical structures, annotated with an asterisk and a number, which were analysed before and after surgery.

Statistical analysis was performed with SPSS (version 28.0.1.0; IBM, Armonk, NY, USA). Normality of the data was assessed using the Shapiro–Wilk test. Normally distributed continuous variables are expressed as mean with standard deviation (SD), otherwise they are expressed as median with interquartile range (IQR). Categorial data are presented as frequencies with percentages. Significance was assessed with the paired samples *t*‐test or the Wilcoxon signed rank test for continuous variables. For the continuous variables acquired from MRD images alpha was adjusted according to the Holm–Bonferroni method [17]. The difference between pre‐ and postoperative position was considered clinically significant when measured as more than 10 mm, since that is the smallest difference that can have clinical implication by affecting the prolapse stage. McNemar's test was used to analyse dichotomous data. Clinical relevance of the PFDI‐20, PFIQ‐7 and PISQ‐12 summary scores was assessed using the minimal important difference (MID)—23, 32 and 6 points, respectively [18, 19].

## RESULTS

The preoperative and postoperative MRD images were acquired and analysed for a total of 21 patients. One patient was unable to comply with the MR protocol due to an inability to defaecate. The results were not discarded, and the maximum strain image of this patient was defined as the frame in which the anorectal junction was at its lowest position. The mean follow‐up time was 9 weeks, ranging from 7 to 13 weeks. Of the 21 patients, one suffered from an external rectal prolapse. Demographic data are summarized in Table 2. Postoperative complications (one bladder lesion with perioperative repair and one mesh erosion without the need for additional treatment during follow‐up) are reported.

**TABLE 2:** Demographic data and surgical complications (*N* = 21 patients).

Variable	Value
Age (years), mean (SD)	63 (11)
BMI (kg/m^2^), mean (SD)	26 (3)
ASA score, *n* (%)	
1	3 (14%)
2	15 (72%)
3	3 (14%)
Number of vaginal deliveries, mean [range]	2 [1–4]
Previous POP surgery, *n* (%)	11 (52%)
Anterior colporrhaphy, *n* (%)	9 (82%)
Posterior colporrhaphy, *n* (%)	7 (64%)
Manchester Fothergill, *n* (%)	1 (9%)
Sacrospinous ligament fixation, *n* (%)	1 (9%)
Hysterectomy to treat POP, *n* (%)	7 (64%)
Previous hysterectomy, *n* (%)	12 (57%)
Episiotomy, *n* (%)	2 (10%)
Perineal tear, *n* (%)	7 (33%)
Perioperative bladder lesion, *n* (%)	1 (5%)
Postoperative mesh erosion, *n* (%)	1 (5%)

Abbreviations: ASA, American Association of Anesthesiologists; BMI, body mass index; POP, pelvic organ prolapse.

In Table 3, the pre‐ and postoperative POP‐Q results are reported. According to POP‐Q staging, a three‐compartment vaginal prolapse was found in two patients (9%), a two‐compartment vaginal prolapse in 10 patients (48%) and a single compartment vaginal prolapse in eight patients (38%); in one patient (5%) no clinical POP was found before surgery. A significant improvement of median POP‐Q stage was found in every compartment. After surgery, no patient suffered from a stage 2 apical or posterior compartment prolapse. In one patient a stage 2 anterior prolapse remained.

**TABLE 3:** Table showing results of the measurements performed on magnetic resonance images, physical examination parameters and PROM scores. A lower or more negative measurement is desired, except for the score of the PISQ‐12 questionnaire. The improvement of all parameters was statistically significant.

Parameter	Presurgery	Postsurgery	*p*‐value
Bladder to PICS^a^, ^c^ (mm) (SD)	22 (22)	8 (9)	0.007
Bladder to PCL^a^, ^c^ (mm) (SD)	29 (22)	16 (9)	0.014
POP‐Q anterior compartment^b^, ^d^ (mm to hymen) [IQR]	−10 [−20–10]	−30 [−30 to –20]	<0.001
VV to PICS^a^, ^c^ (mm) (SD)	6 (23)	−38 (17)	<0.001
VV to PCL^a^, ^c^ (mm) (SD)	21 (22)	−18 (17)	<0.001
POP‐Q apical compartment^b^, ^d^ (mm to hymen) [IQR]	−40 [−50 to 0]	−70 [−80 to –70]	<0.001
ARJ to PICS^a^, ^c^ (mm) (SD)	41 (13)	36 (13)	0.013
ARJ to PCL (M‐line)^a^, ^c^ (mm) (SD)	68 (11)	62 (11)	0.002
POP‐Q posterior compartment^b^, ^d^ (mm to hymen) [IQR]	0 [−20 to 10]	−30 [−30 to –30]	<0.001
H‐line^b^, ^c^ (mm) [IQR]	99 [91–109]	92 [84–102]	0.004
Rectocele size^b^, ^c^ (mm) [IQR]	35 [29–40]	19 [18–23]	<0.001
Enterocele size^a^, ^c^ (mm) (SD) *n* = 9	42 (24)	−12 (26)	<0.001
PFDI‐20^a^, ^d^ score (SD)	135 (52)	44 (26)	<0.001
PFIQ‐7^a^, ^d^ score (SD)	98 (50)	29 (33)	<0.001
PISQ‐12^a^, ^d^ score (SD)	28 (7)	33 (5)	0.004

Abbreviations: ARJ, anorectal junction; IQR, interquartile range; PCL, pubococcygeal line; PFDI‐20, Pelvic Floor Disability Index‐20; PFIQ‐7, Pelvic Floor Impact Questionnaire‐7; PICS, pelvic inclination correction system; PISQ‐12, Pelvic Organ Prolapse/Urinary Incontinence Sexual Questionnaire‐12; POP‐Q, Pelvic Organ Prolapse Quantification; PROM, patient reported outcome measure; VV, vaginal vault;

^a^ Continuous variable reported as mean and SD and significance tested using the paired *t*‐test.

^b^ Continuous variable reported as median and IQR and significance tested using the Wilcoxon signed rank test.

^c^ Significance tested with adjusted *α* according to the Holm–Bonferroni method.

^d^ Significance tested with *α* = 0.05.

Based on upright MRD a significant lift in organ position was found for the bladder (14 mm, *p* = 0.007), VV (44 mm, *p* < 0.001) and ARJ (5 mm, *p* = 0.013), and a size reduction of the M‐line (6 mm, *p* = 0.002), H‐line (7 mm, *p* = 0.004), the rectocele (16 mm, *p* < 0.001) and the enterocele (54 mm, *p* < 0.001) during defaecation.

The PROMs show a significant improvement in all summary scores and subscales. For the PFDI‐20 (*p* < 0.001), PFIQ‐7 (*p* < 0,001) and PISQ‐12 (*p* < 0.001), 18 patients (86%), 16 patients (76%) and 4 patients (44%), respectively, reported an improvement larger than the MID. A significant decrease in symptoms of ODS and FI (based on the PFDI‐20 questionnaire), except incontinence for soft defaecation, was found as shown in Table 4.

**TABLE 4:** Results of Pelvic Floor Disability Index‐20 questions specific for FI or ODS.

Symptom		Preoperative, *n* (%)	Postoperative, *n* (%)
ODS	Sensation of incomplete evacuation^a^	20 (95%)	12 (57%)
ODS	Excessive straining during evacuation^a^	18 (85%)	5 (24%)
ODS	Need for digitation to evacuate stool^a^	16 (76%)	4 (19%)
FI	Uncontrollable flatulence^a^	18 (85%)	11 (52%)
FI	Incontinence for soft stool	11 (52%)	5 (24%)
FI	Incontinence for normal stool^a^	11 (52%)	2 (10%)

Abbreviations: FI, faecal incontinence; ODS, obstructed defaecation syndrome.

^a^ Statistically significant with *α* = 0.05.

**FIGURE 2:**
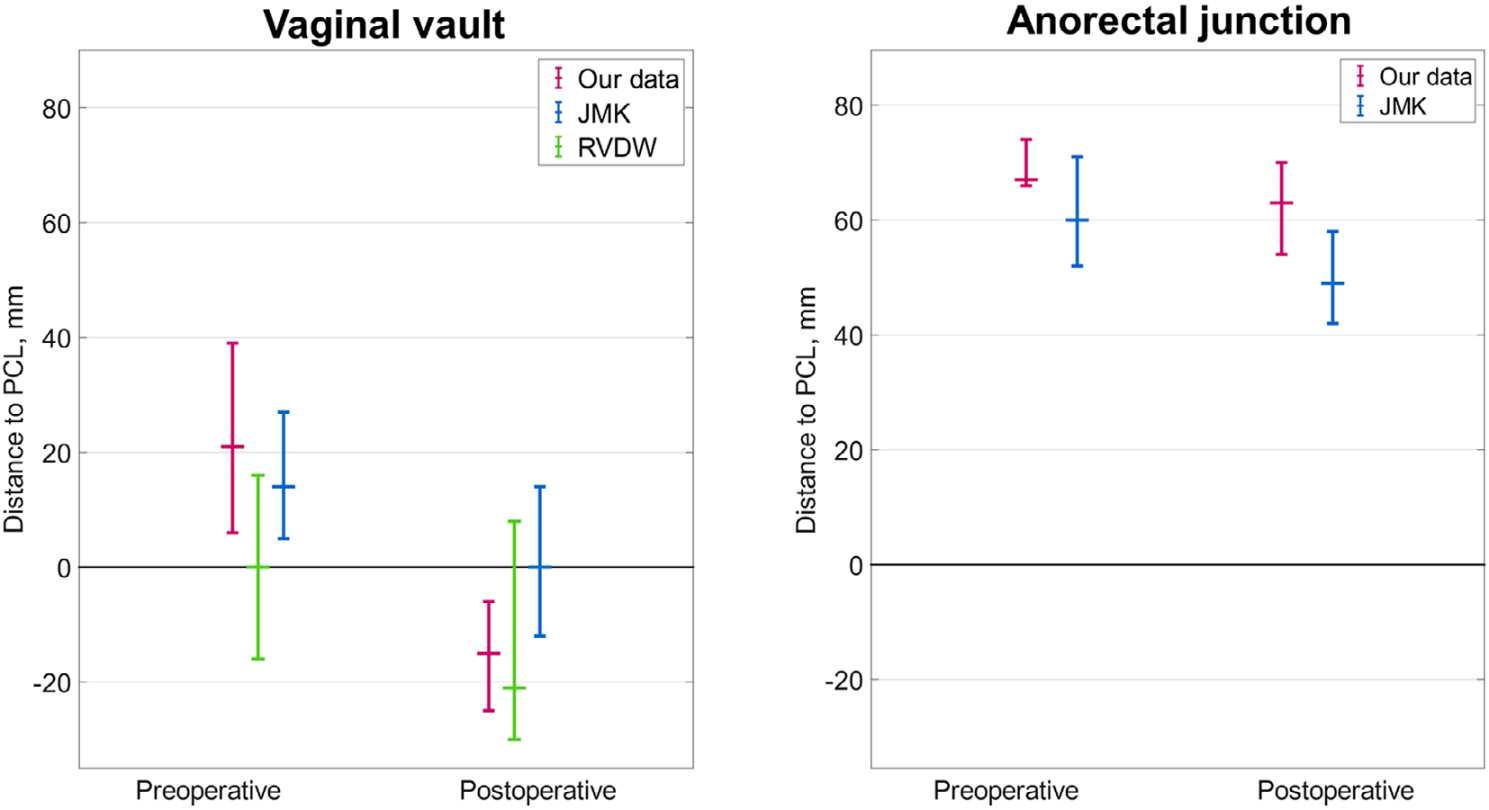
Results for the position of the vaginal vault (left) and anorectal junction (right) compared with studies performed using supine imaging by Mäkelä‐Kaikkonen et al. (JMK) and Van der Weiden et al. (RVDW) [13, 14]. The upper and lower boundary of the error bar represents the interquartile range and the marker represents the median. PCL, pubococcygeal line.

Figure 2 compares our measurements of the VV and ARJ and values reported in the literature [13, 14]. In the left graph upright analysis shows a larger lift, indicated by a larger difference in preoperative versus postoperative VV position after surgery. Supine data from the standalone VMR [14] is comparable to the data from the standalone SCP [13]. In the right graph of Figure 2 upright imaging shows a larger descent of the ARJ, indicated by a larger distance to the PCL, in both pre‐ and postoperative measurements when compared with standalone VMR studied in supine position.

## DISCUSSION

The aim of this research was to identify the change in pelvic anatomy after SCRP by means of upright MRD. In a population of 21 patients with proven IRP or ERP and POP, upright MRD assessment shows that the position of the bladder, VV and ARJ, the length of the M‐line and H‐line, and the size of the enterocele and rectocele have been significantly improved by the surgery. The improvements for the bladder, VV, rectocele and enterocele are considered clinically relevant as the postoperative improvement is more than 10 mm. Compared with previous supine imaging studies, upright imaging shows a larger organ lift for the VV as well as a higher median value of the ARJ to the PCL in both pre‐ and postoperative measurements.

The main strengths of this research are the prospective design of the study and the use of distance measurements on imaged anatomy. To the best of our knowledge no previous studies have reported these types of data on combined SCRP surgery, neither in supine nor upright position. Previous studies on the effect of surgery are based on physical examinations and questionnaires and followed a retrospective design [20, 21].

This study has two main limitations: first the short follow‐up time of 7–13 weeks postsurgery. The results of this research depict the immediate postoperative situation during which patients are instructed to strictly limit the forces on their pelvic floor (e.g. no heavy lifting or pelvic pushing). This study thus illustrates the most optimal effect of SCRP, shortly after surgery, in the position of normal daily activity (upright position). Further follow‐up is recommended to assess the pelvic anatomy at a timepoint when the healing process has ended and patients have resumed their normal activities.

The second limitation is the limited sample size. Due to the exploratory character of this study only 21 patients were included in the analysis. This means that the conclusions from these data might not be generalizable to the full population. The results, however, indicate interesting directions for further research.

This study is the first to assess pelvic organ position after SCRP using upright MRD. However, there are studies that performed similar measurements using supine MRD for assessment of standalone VMR and standalone SCP [13, 14].

The first parameter that can be compared is the VV lift. Comparison of the position of the VV with respect to the PCL reference line before and after surgery shows a larger effect of surgery than supine imaging. Previous studies suggest that supine imaging leads to an underestimation of POP and IRP [10, 12]. Thereby, supine assessment is commonly done with the patient straining to mimic the anatomy in upright position. A preoperative patient might also be unwilling to strain at maximum capacity due to the fear of urinary or faecal incontinence. Furthermore, a postoperative patient, who has been instructed to avoid or limit abdominal pressure for 6 weeks, might be hesitant to perform Valsalva out of fear of prolapse recurrence.

A second parameter that can be compared with previous literature is the position of the ARJ (Figure 2, right graph). Although the slope of both lines is similar, the descent of the ARJ is larger in the upright position both before and after surgery. Following the hypothesis that functional improvement is linked to ARJ descent, an accurate assessment of the amount of ARJ descent could be relevant to understand the effect of improvement in PROMs after surgery.

According to the PROMs an overall improvement in symptoms, impact on quality of life and sexual function can be observed in the studied population. Eighteen of the 21 patients (90%) showed a score improvement larger than the MID for PFDI‐20. Additionally, two patients still scored higher than the patient‐accepted symptom score (PASS), set at 60 [22]. This suggests that for these patients the surgical intervention did not have the desired effect on the symptoms. Five patients indicated, based on the PFIQ‐7, that there was no relevant improvement in the impact of symptoms on their social life. One patient even reported a worsening of the effect of the symptoms on their social life. Three patients who showed no relevant improvement had a PFIQ‐7 score of less than 62 out of 300. This suggest that the initial impact on symptoms was low, reducing the chance that the MID requirement is met. The PISQ‐12 has the lowest response rate (*n* = 9), possibly due to the instruction not to have penetrative sex in the 6 weeks after surgery. PISQ‐12 results are therefore less reliable in the current study and should be repeated at a later follow‐up moment.

After SCRP, a significant reduction of all symptoms, except FI for soft defaecation, was achieved (Table 4). However, when looking at the individual responses it can be noticed that specific symptoms remain in 10%–57% of the patients. This underlines the essence of understanding the effect of surgery on functional outcome. Investigating the relation between the PROMs and patient anatomy is beyond the scope of this article. However, for future research it would be interesting to analyse the anatomy, function and symptoms of unsatisfied and satisfied patients on an individual level in a larger cohort and with a longer follow‐up period.

Our hypothesis, that most of the anatomical change is visible in structures that are directly attached to the mesh implants during surgery, is only partially confirmed. A significant improvement in the position of the ARJ, M‐line length and H‐line length was found. As the mesh was not attached to the pelvic floor or anal sphincter a significant improvement was not expected. These improvements indicate a lift of the anus and shortening of the hiatus, suggesting that pelvic floor relaxation has been reduced compared with the preoperative situation [23]. At this time, the limited sample size and short follow‐up period limit the possibility of correlating anatomical change to functional outcome. Future research should include an objective measure for pelvic floor function, such as 3D transperineal ultrasound [24].

Assessment of the pelvic anatomy after SCRP by means of upright MRD shows, in contrast to standalone VMR or SCP, that there is a clinically and statistically significant improvement in the prolapse of the bladder, VV, rectocele and enterocele. Upright MRD additionally shows pre‐ and postoperative differences in pelvic anatomy compared with supine measurements. Therefore, upright assessment might be essential in developing an accurate understanding of the (limited) improvement in functional outcome after SCRP.

## AUTHOR CONTRIBUTIONS

**Mart C. P. Kortman:** Methodology; investigation; formal analysis; writing – original draft; software; data curation. **Jan W. P. Vanstiphout:** Writing – review and editing; investigation; methodology; conceptualization. **Akeel Alhafidh:** Writing – review and editing; investigation; methodology; conceptualization. **Frank F. J. Simonis:** Writing – review and editing; supervision; methodology; conceptualization. **Anique T. M. Grob:** Writing – review and editing; funding acquisition; supervision; methodology; conceptualization.

## FUNDING INFORMATION

This research was supported by the TORBO grant based on the local hospital ZGT science voucher (ACA0022) and the SMEAR grant based on the local Pioneers in Healthcare grant (PIHC‐2023).

## CONFLICT OF INTEREST STATEMENT

None of the authors report a conflict of interest or a relevant financial relationship.

## ETHICS STATEMENT

The study protocol was approved by a medical ethics committee and registered as NL79717.091.21.

## PATIENT CONSENT STATEMENT

All patients provided written informed consent.

## Data Availability

The data that support the findings of this study are available on request from the corresponding author. The data are not publicly available due to privacy or ethical restrictions.
